# Wellens Syndrome: An Atypical Presentation With Burning Chest and Epigastric Pain

**DOI:** 10.7759/cureus.16241

**Published:** 2021-07-07

**Authors:** Sunil Shah, Amit K Jaiswal, Nischal Neupane, Divya Karki

**Affiliations:** 1 Medicine, Ministry of Health, Malé, MDV; 2 Medicine, California Institute of Behavioral Neurosciences & Psychology, Fairfield, USA; 3 Internal Medicine, Ministry of Health, Malangwa, NPL; 4 Pediatrics, Ministry of Health, Malé, MDV

**Keywords:** inverted t-waves, biphasic t-waves, left anterior descending artery, wellens syndrome, intermittent chest pain

## Abstract

Wellens syndrome is a pre-infarction stage due to the critical stenosis of the proximal left anterior descending artery. It is characterized by intermittent chest pain with classical ECG changes. The cardiac biomarker is within the normal limit or only slightly elevated in this condition. Early recognition and cardiac intervention are important to prevent adverse cardiac outcomes. We report this rare case of Wellens syndrome in a 70-year-old male with intermittent chest and epigastric pain associated with belching for five days. The patient presented characteristic T-wave changes, symmetric deeply inverted T-waves, in precordial leads (V2-V4). Cardiac biomarkers, including troponin, were negative. He underwent cardiac catheterization and found a clot in the proximal left anterior descending coronary artery, which required a catheter-directed thrombectomy and drug-eluting stent placement. It is important to recognize this condition early and referred on time from primary health centers to higher centers, especially in developing countries like the Maldives, since delays in transfer may lead to a serious outcome.

## Introduction

Wellens and his colleagues first described the clinical features and ECG criteria of a sub-group of patients with myocardial ischemia that later got its name, Wellens syndrome [1]. It is also referred to as left anterior descending coronary (LADC) T-wave syndrome since the typical ECG pattern suggests the high-grade blockade of the LADC artery (LADCA) [2]. The distinctive feature of this syndrome is chest pain with characteristic electrocardiographic changes described as biphasic or deeply inverted T-waves in the anterior chest leads [3]. These changes can be missed in the Emergency Department leading to myocardial infarction (MI) of the major anterior ventricular muscle and the septum. Subsequently, this can lead to heart failure and other complications such as arrhythmia and death. Thus, it is very important to diagnose and manage this case during the initial presentation to prevent adverse clinical outcomes. Here, we present this case in a 70-year-old male with a burning chest and epigastric pain referred from a primary health center on an outlying island to our hospital in the Maldives.

## Case presentation

A 70-year-old male was presented to our hospital complaining of on and off chest pain for five days. The pain was over the epigastrium and the left side of the chest with no radiation. It was burning in character and associated with epigastric discomfort and belching. There was no associated sweating, breathing difficulty, abdominal pain, headache, fever, swelling of legs, and any changes in appetite and weight. There were histories of hypertension and hyperlipidemia for which he was on amlodipine 5 mg daily and atorvastatin 20 mg daily, respectively. He had a history of gastroesophageal reflux disease. There was a 30-pack-year of smoking history, and he denied intake of alcohol or any illicit drugs.

**Figure 1:**
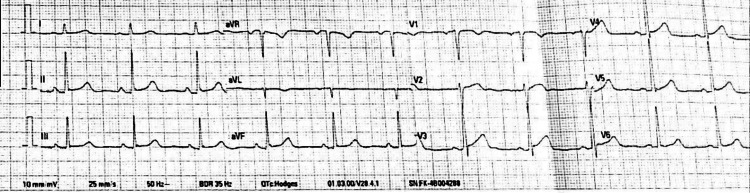
ECG at Initial Presentation to the Health Center aVL - augmented Vector Left; aVF - augmented Vector Foot

For these complaints, he had initially visited the local health center. Antacid syrup and intravenous (IV) pantoprazole were given, and an ECG was done. The initial ECG done in the local health center is as shown in Figure 1.

**Figure 2:**
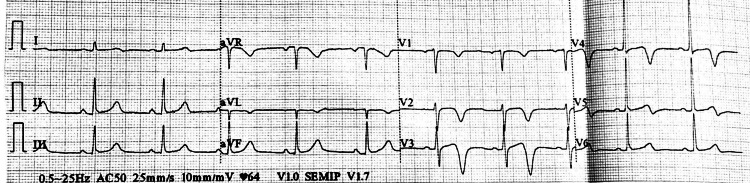
Characteristic T-Wave Changes in V2-V4

With these complaints and ECG findings, he was referred to our hospital. At presentation, his vitals were stable, and saturation was normal on room air. The lab results revealed a normal complete blood count and comprehensive metabolic panel with negative troponin-T, CK-MB 15 U/L (reference range 0-24 U/L), and troponin-I 0.009 ng/mL (reference range < 0.03). The chest x-ray was normal, and the ECG taken at our hospital showed deeply inverted T-waves in anterior chest leads (V2-V4), as shown in Figure 2 suggesting Wellens syndrome type II.

He was treated medically with one dose of 300 mg aspirin followed by 75 mg aspirin daily, atorvastatin 40 mg daily, and started on a heparin drip. A transthoracic echocardiogram of the heart was done, and he underwent cardiac catheterization. He was found to have a clot in the proximal LADCA, which required catheter-directed thrombectomy and drug-eluting stent placement. He did well throughout the procedure, and his vitals were stable after the procedure.

## Discussion

Wellens syndrome results from a temporary obstruction of the LADCA with a high risk of progression to MI; thus, it represents a pre-infarction stage. There are normal to minimally elevated cardiac enzymes with no significant ST elevation or Q-waves in Wellens syndrome [1]. The ECG changes are characterized by normal precordial R-wave progression and characteristic T-wave changes [1,4]. These findings suggest a high-grade blockade of the proximal LADCA, hence, the name LAD coronary T-wave syndrome. Approximately 75% of patients develop acute anterior wall MI within a week if the timely intervention is not done [1,5].

The risk of an extensive MI of the anterior wall due to complete LADCA blockade can be prevented by early recognition of the typical ECG pattern of Wellens syndrome. There are two subtypes of this disease depending on patterns of T-wave abnormalities in precordial leads. Type A is characterized by biphasic T-wave and is more common (nearly 75% of total cases), and type B, the less common type (nearly 25% of the cases), is characterized by deeply inverted T-waves in V2-v4 [6,7]. With reperfusion therapy done for MI/ischemia, there is a recurrence of biphasic T-wave; thus, the pathophysiology of the T-wave changes can be related to spontaneous reperfusion following occlusion of the LADCA [8]. The risk factors of this disease are similar to the risk factors of other coronary artery diseases such as hypertension, diabetes mellitus, hyperlipidemia, those with a family history of premature heart disease, metabolic syndrome, personal history of smoking, occupational stress, and sedentary lifestyle.

The clinical features of Wellens syndrome may be mistaken for chronic stable angina as the chest pain occurs intermittently, and the symptoms can develop over days to weeks. There can be ST elevation during the chest pain while there are characteristic ECG changes during a pain-free period, as shown in Table 1 [7]. This leads to see T-wave changes in Wellens syndrome are V2 and V3 corresponding to a regional supply of first and second septal branches of the LADCA, although it can be seen along with the precordial leads widely if the lesion is more proximal in the LADCA [6]. The cardiac biomarkers, including troponin, may be normal or mildly elevated.

 

**Table 1:** Clinical Features of Wellens Syndrome mm = millimeters

Intermittent chest pain
Normal or slightly increased level of cardiac enzymes
No precordial Q-waves or poor R-wave progression.
Deeply inverted symmetrical T-waves or biphasic T-waves in v2-v5 or v6 during pain-free period
Minimal ST elevation or depression (<1 mm)

The typical ECG pattern has a sensitivity and specificity value of 69% and 89%, respectively, and a positive predictive value of 86% [9]. Emergency angiography is recommended in both types of ECG changes [1,4]. The differential diagnoses include left ventricular hypertrophy, bundle branch blocks, aortic dissection, tension pneumothorax, pulmonary embolism, Takotsubo syndrome, digitalis use, and cocaine-induced vasospasm [10-12]. Wellens syndrome usually requires invasive management with cardiac catheterization and subsequent angioplasty or coronary bypass grafting as it does not respond well to medical treatment only [1,4].

## Conclusions

Timely recognition of Wellens syndrome by characteristic T-wave changes is critical. The diagnosis of this disease can be missed or misdiagnosed as gastritis/gastroesophageal reflux disease, causing a delay in transferring the patient to a higher center where cardiac catheterization is available, especially in developing countries like the Maldives, which lack tertiary care facilities in village areas. Thus, prompt diagnosis and consultation with cardiologists, including early referral from the local health center, are required to prevent adverse cardiac outcomes such as cardiac failure and death due to acute MI.
